# Correction: Toward an understanding of the structural basis of allostery in muscarinic acetylcholine receptors

**DOI:** 10.1085/jgp.20171197912022024c

**Published:** 2024-12-19

**Authors:** Wessel A.C. Burger, Patrick M. Sexton, Arthur Christopoulos, David M. Thal

Vol. 150, No. 10 | https://doi.org/10.1085/jgp.201711979| September 06, 2018

In this Viewpoint, the clinical trial of xanomeline for treating Alzheimer’s disease, referencing Bodick et al., 1997 (Effects of xanomeline, a selective muscarinic receptor agonist, on cognitive function and behavioral symptoms in Alzheimer disease. Arch Neurol. 54:465–473), was mistakenly described as a phase 3 trial.

Xanomeline was the first molecule developed by Lilly for the treatment of Alzheimer’s disease; the program was discontinued following a phase 2 trial (https://www.lilly.com/discovery/alzheimers-disease/timeline).

The corrected text is shown in bold:

“For example, the M_1_/M_4_ mAChR-preferring agonist, xanomeline, showed promising results in a **phase 2** Alzheimer’s disease trial (Bodick et al., 1997) and a small proof-of-concept trial in schizophrenia (Shekhar et al., 2008) but was ultimately abandoned because of unacceptable side effects, including those mediated by the related M_2_ and M_3_ subtypes in the periphery (Kruse et al., 2014).”

The errors appear in print and in PDFs downloaded before December 3, 2024.
